# The Biological Properties of OGI Surfaces Positively Act on Osteogenic and Angiogenic Commitment of Mesenchymal Stem Cells

**DOI:** 10.3390/ma10111321

**Published:** 2017-11-17

**Authors:** Paolo Ghensi, Eriberto Bressan, Chiara Gardin, Letizia Ferroni, Maria Costanza Soldini, Federico Mandelli, Claudio Soldini, Barbara Zavan

**Affiliations:** 1 Department of Neurosciences, Dental School, University of Padova, Via Giustiniani 2, 35100 Padova, Italy; dr.ghensi@gmail.com (P.G.); eriberto.bressan@unipd.it (E.B.); claudiosoldini.it@gmail.com (C.S.); 2 Centre for Integrative Biology (CIBIO), University of Trento, 38122 Trento, Italy; 3 Department of Biomedical Sciences, University of Padova, Via Ugo Bassi 58, 35100 Padova, Italy; chiara.gardin@unipd.it (C.G.); letizia.ferroni@unipd.it (L.F.); 4 Department of Public Health, Experimental and Forensic Medicine, University of Pavia, 27100 Pavia, Italy; costanzasoldini@hotmail.it; 5 Private Practice, 09100 Milano, Italy; federico.mandelli@gmail.com; 6 CLC Scientific, via Vecchia Ferriera 18, 36100 Vicenza, Italy; 7 Maria Cecilia Hospital, GVM Care & Research, 48033 Cotignola (RA), Italy

**Keywords:** titanium surface, dental implant, MSC

## Abstract

Osteogenesis process displays a fundamental role during dental implant osteointegration. In the present work, we studied the influence of Osteon Growth Induction (OGI) surface properties on the angiogenic and osteogenic behaviors of Mesenchymal Stem cells (MSC). MSC derived from dental pulp and HUVEC (Human Umbilical Vein Endothelial Cells) were grown in on OGI titanium surfaces, and cell proliferation and DNA synthesis were evaluated by MTT [3-(4,5-dimethylthiazol-2yl)-2,5-diphenyltetrazolium bromide] test and DNA quantification. Gene expression has been performed in order to evaluate the presence of mRNA related to endothelial and osteogenesis markers. Moreover, morphological and biochemical analyses of osteogenesis commitments has been performed. On OGI surfaces, MSC and HUVEC are able to proliferate. Gene expression profiler confirms that MSC on OGI surfaces are able to express endothelial and osteogenic markers, and that these expression are higher compared the expression on control surfaces. In conclusion On OGI surfaces proliferation, expression and morphological analyses of angiogenesis-associated markers in MSC are promoted. This process induces an increasing on their osteogenesis commitment.

## 1. Introduction

An adequate amount of bone tissue surrounding dental implants is fundamental to reach a satisfactory treatment outcome in the long-term period [1,2,3]. Therefore, there is a need to further improve the performance of the implants particularly in a bad bone conditions and to optimize the time of osteointegration process [4,5,6].

Osteointegration is defined as a “direct structural and functional connection between ordered living bone and the surface of a load-carrying implant”. Although osteointegration was meant originally to describe a biologic fixation of the titanium dental implants, it is now used to describe the attachment of other materials used for dental and orthopedic applications as well. Analyses of material-bone interface showed that osteointegrated implants can have an intervening fibrous layer or direct bone apposition characterized by bone-bonding depending on the composition and surface properties of the biomaterial. Research on dental implant designs, materials, and techniques has increased in the past few years and is expected to expand in the future due to the recent growth of the global market for dental implants and the rising in the demand for cosmetic dentistry.

In order to increase the success rate of dental implants, research has focused on the control of surface properties such as morphology, topography, roughness, chemical composition, surface energy, residual stress, the existence of impurities, thickness of Ti oxide film, and the presence of metallic and nonmetallic compounds on the surface. These properties profoundly influence the osseous and tissue response to the implant by either increasing or decreasing healing times and osseointegratio. Research has shown that osteoblastic cells adhere more quickly to rough surfaces than to smooth surfaces1. This property can also produce orientation and guide locomotion of specific cell types and has the ability to directly affect cell shape and function.

The biological events during the early step of osteointegration are directly influenced by the osseous microenvironment (i.e., cells, matrix and signaling molecules) into which the implant is inserted and have several analogies with general wound healing mechanisms [7,8,9,10,11,12]. From a clinical point of view, it is observed that the neo vascularization of the implant bed plays a key-role in bone tissue formation and this dynamic process is the result of complex biological phases [13,14,15,16]. It is demonstrated that the first days of healing are crucial for the survival of the implant osteointegration whereby bone formation and remodeling occur in parallel around the implant [17,18,19,20].

Angiogenesis, the formation of new blood vessels from pre-existing capillaries, has a significant effort to inflammatory and regenerative processes in the body’s tissues including bones [21,22]. Blood vessels are the fundamental component of bone formation, and maintenance and formation of an adequate vascular bed is a key-factor to support the metabolic needs of the newly forming tissues [23,24]. Consequently, it is evident how angiogenesis temporally precedes osteogenesis. In fact the formation of new bones, bone modeling and remodeling and also osteointegration after dental implant placement need a blood supply to guarantee nutrition, oxygen, and osteoprogenitor cells through the newly formed blood vessels [25].

The primary cells implicated in angiogenesis are endothelial cells. They are involved on the realization of the microvasculature that is responsible for the supply of nutrients and oxygen and for the elimination of the waste. Moreover they participate in the tissue response thanks to expressing of osteoblast adhesion molecules and by the releasing of pro-inflammatory factors [26]. In the end there is an intimate connection between endothelial cells and bone cells, and information of blood vessels in bone enhances osteogenesis that takes place in the neighborhood of newly formed vessels [27]. Several titanium surfaces are available, and in this scenario in the present work we have better characterized the biological properties of a novel dental surfaces OGI that we have previously characterized [28] by mechanical point of view. OGI implants are characterized by a large-grit sandblasted and acid etched surface (Ra. 0.31–1.61). SEM(scanning electron microscope ) and XPS (X-ray photoelectron spectroscopy) analyses have previously demonstrated this where the presence of both big “holes” due to large grit sandblasting on which the micro roughness due to acid etching is superimposed has been confirmed. Moreover, tridimensional topography, which imparts to these surfaces spongy-like characteristics, has been confirmed thanks to the short peak-to-peak distance, on the order of about 1 μm. According to this close relationship between osteogenesis, angiogenesis, and osteointegration, the aim of the present study is to demonstrate through molecular, biochemical, and morphological analysis how an Osteon Growth Induction (OGI) titanium implant surface is able to own angiogenic properties that positively act on osteogenic commitment of mesenchymal stem cells (MSCs) and consequently on the osteointegration process.

## 2. Results

### 2.1. Proliferative Activity 

**Figure 1:**
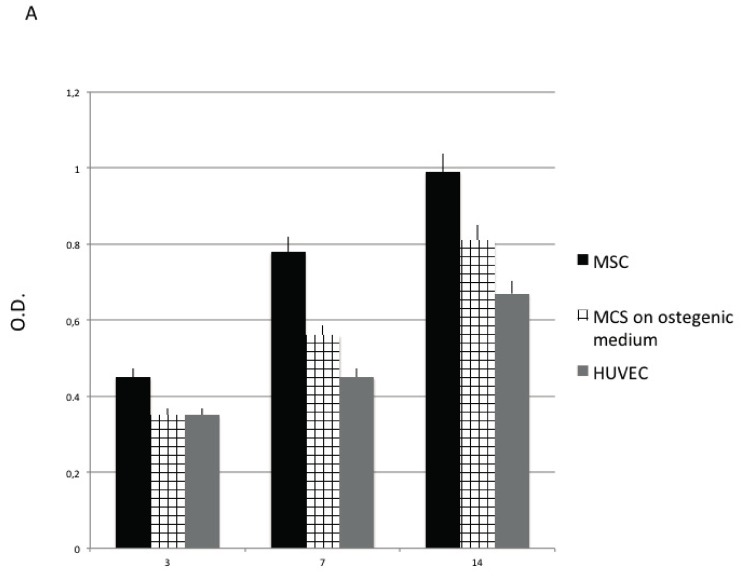

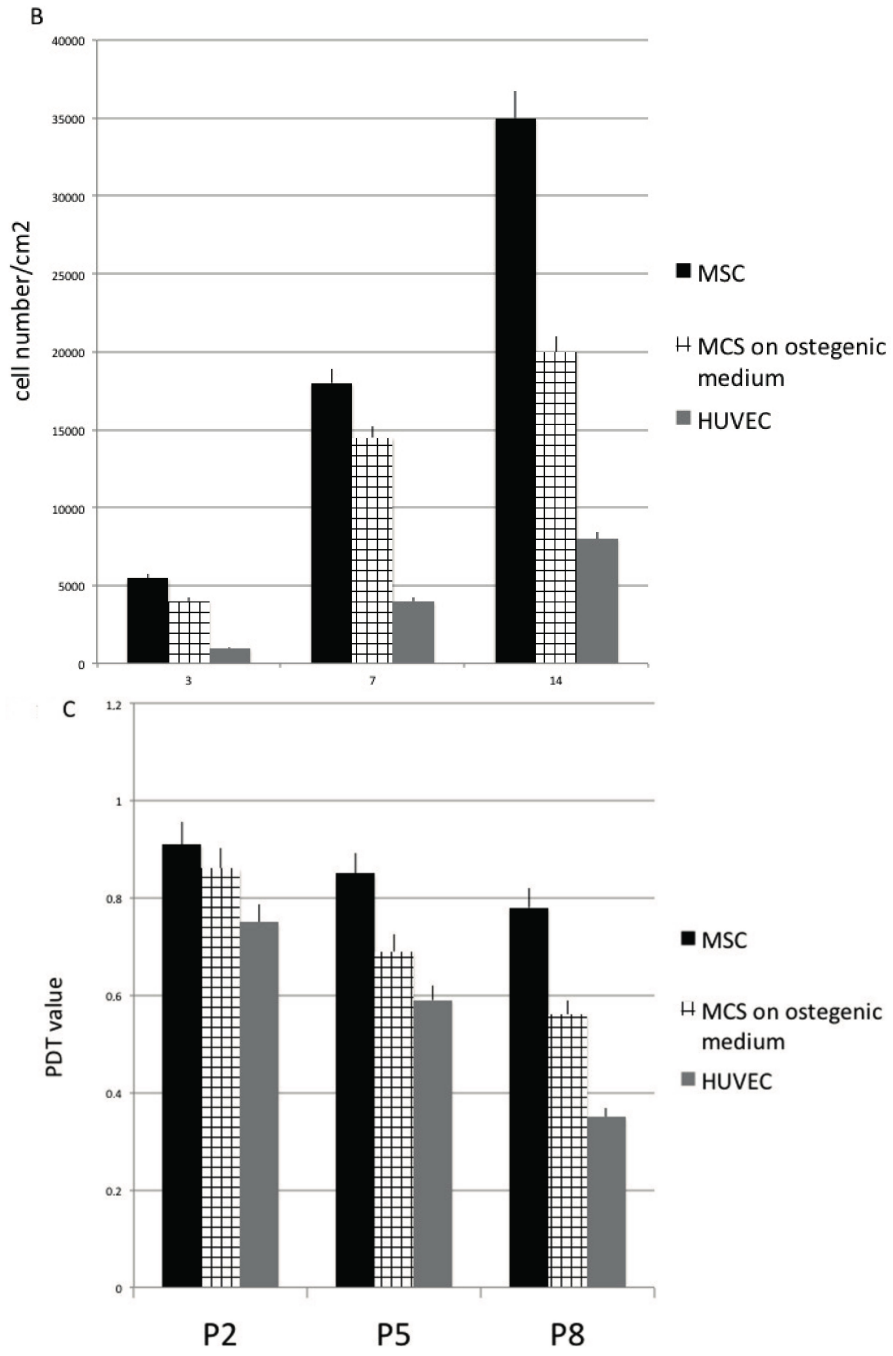
Proliferative activity of Mesenchymal Stem cells (MSC) detected by means of MTT test (**A**), DNA content (**B**) population doubling time (PDT) value (**C**). MSC cultured in presence of nondifferentiative medium: (black bars), osteoinductive medium (squared bars), HUVEC (Human Umbilical Vein Endothelial Cells) (grey bars.).

In Figure 1, proliferative activity of MSC (in the presence of nondifferentiative medium), MSC cultured in the presence of osteoinductive medium (representing here osteoblastic like cell population) and HUVEC (representing here endothelial cells population) have been detected employing MTT test, DNA content, and population doubling time (PDT) value.

As reported in Figure 1C, a well-defined cell growth on the OGI surfaces for each passage for each cells group is observed. The cells are able to well proliferate, increasing in number as demonstrated by the MTT test Figure 1A and the DNA content (Figure 1B). The rising MTT values confirmed that the cells were alive when cultured onto OGI surfaces, whether their ability to proliferated is confirmed by the increase in DNA content. These dates are also supported by the population doubling time (PDT) that is a direct marker of the proliferative ability of the cell since it is used to evaluate the ability of the cell to duplicate in number. In our experiment, we analyzed the PDT of the different cell populations at three different time: 3, 7 and 14 days.

As reported in Figure 1C, it is observed a well-defined cell growth for each passage for each cells group. When the cells are in a mesenchymal stage (MSC) a high PDT value was maintained in all cell passages confirming the right proliferative ability of the cells when are in a stem cells phenotype. For the other cells population (related to a committed one), both osteoblastic-like and endothelial one (HUVEC) proliferative ability decreased in time and during in vitro commitment.

### 2.2. Endothelial Cells 

**Figure 2:**
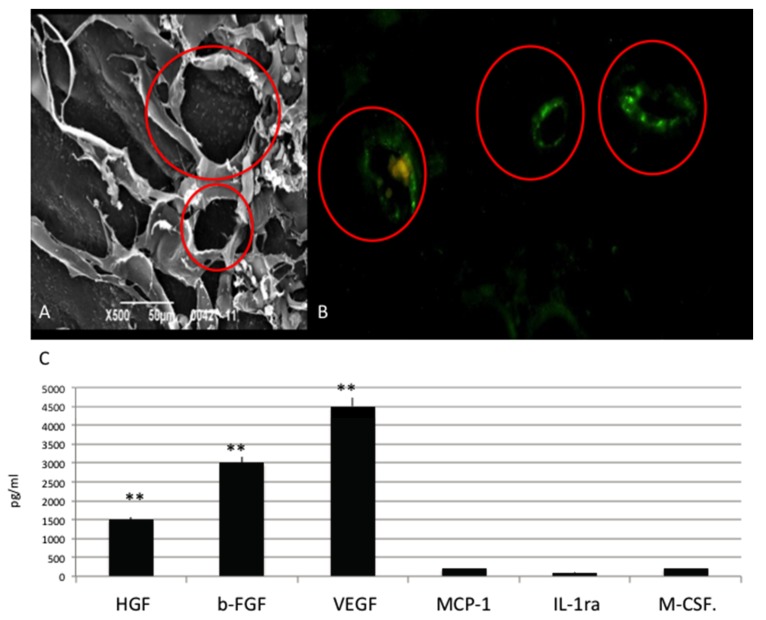
(**A**) SEM(scanning electron microscope ) analyses of HUVEC cultured onto Osteon Growth Induction (OGI) surfaces; (**B**) immunoistochemical test against CD31 (in green), capillary like structures (red circles) as SEM analyses revealed; (**C**) Quantification of growth factor release pg/mL. Statistically significant differences are indicated as ** *p* < 0.01.

To determine how OGI features affect the morphology of HUVECs, HUVECs were analyzed for morphological responses Figure 2 after 14 days of culture. Our results show that on OGI surfaces HUVECs were able adhere and to spread well. Cells were flattened with pronounced protrusion of philopodia. Cells were able to attach tightly to OGI surfaces and covered a great area. This has been confirmed by means of the morphology, by the enhancement of cell-cell communication, and by the cell-substrate adhesion. In Figure 2A SEM test and in Figure 2B immunoistochemical test against CD31 (in green) clearly show that cells can attach onto the surface organizing in capillary like structures (red circles). Maintaining of the correct phenotype also confirmed by the presence of CD31 receptor (typical of mature endothelial cells) onto the surface (in green). As reported in Figure 2, HUVEC are able to attach to the OGI surface and organized themselves into capillary like structures (red circles), as SEM analyses revealed. Positivity for CD31 (in green) confirms that HUVEC can maintain their endothelial phenotype.

In Figure 2C results related to ELISA (enzyme-linked immunosorbent assay) assay direct to evaluate quantitatively the secretion of the VEGF and bFGF proteins over 14 days are reported. For VEGF and bFGF protein secretion, it is clear that their level are significantly higher compared to the other protein secreted typical of a non-endothelial phenotype.

### 2.3. Osteoblastic Like Cells

**Figure 3:**
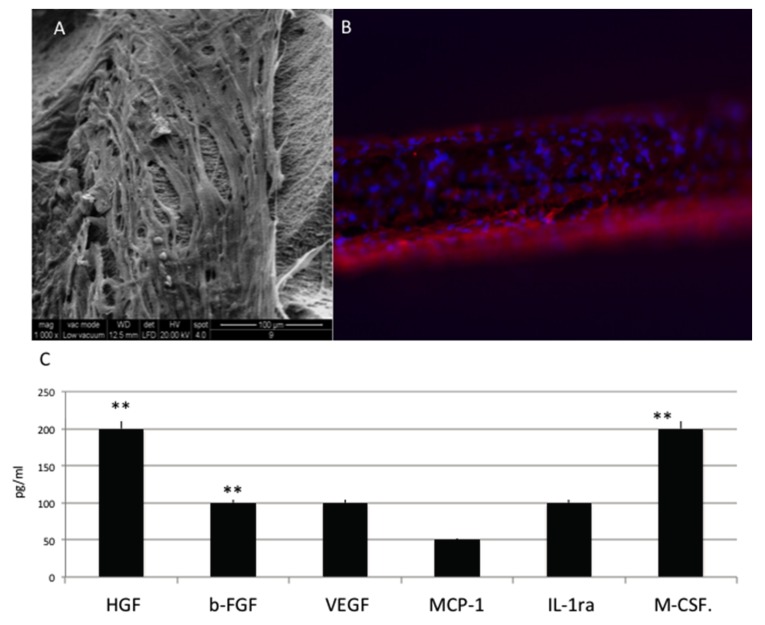
(**A**) SEM analyses of MSC in presence of osteoinductive medium cultured onto OGI surfaces; (**B**) immunohistochemical test against phalloidine (red) (**C**) Quantification of growth factor release pg/mL. Statistically significant differences are indicated as ** *p* < 0.01.

When MSC are cultured into osteogenesis medium are able to attach into all the surfaces as electro-microscopy (Figure 3A) and immunoistochemical analyses for phalloidine (Figure 3B in red) analyses confirm. Cell spread well and colonized all the surface. Well-defined phyllopodia, ensuring the attached, are well evident by the positivity for phalloidin (in red. Figure 3B) shows.

To assess differences between HUVEC, MSC and osteoblastic like cells regarding the release of paracrine factors with several supportive effects such as antiapoptotic, immunomodulatory, antifibrotic, angiogenic, chemoattractive, and hematopoiesis, the following factors were quantified: Hepatocyte growth factor (HGF), basic fibroblast growth factor (b-FGF), vascular endothelial growth factor (VEGF), monocyte chemotactic protein-1 (MCP-1), stromal cell-derived factor 1-alpha (SDF-1a), interleukin 1 receptor antagonist (IL-1ra) and macrophage colony-stimulating factor (M-CSF) (Figure 3C). In this cells cultures, lower factors related to endothelial phenotype such as VEGF and bFGF, whether typical markers of osteoblast such as Hepatocyte growth factor (HGF) and colony stimulating factor 1 (M-CSF), are significantly expressed.

Cells of mesenchymal origin, and osteoblasts/osteocytes and bone marrow stromal cells originate from mesenchymal cells are able to mainly produce HGF. M-CSF is released by osteoblasts and exerts effects on osteoclasts thank to its paracrine effect. M-CSF binds to receptors on osteoclasts are able to induce differentiation, and to lead the increasing of plasma calcium levels.

### 2.4. MSC

**Figure 4:**
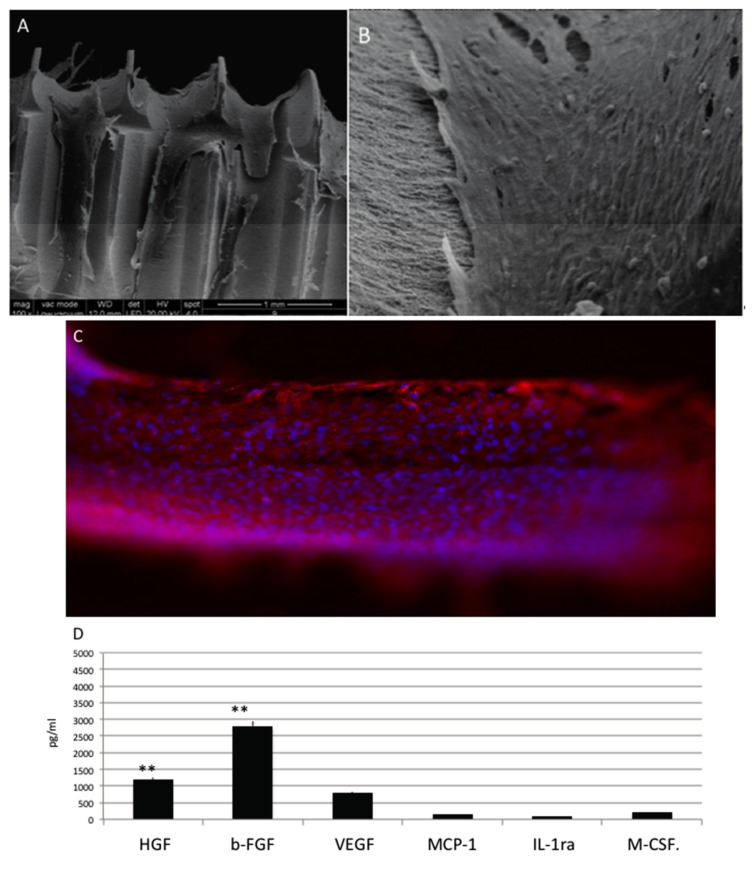

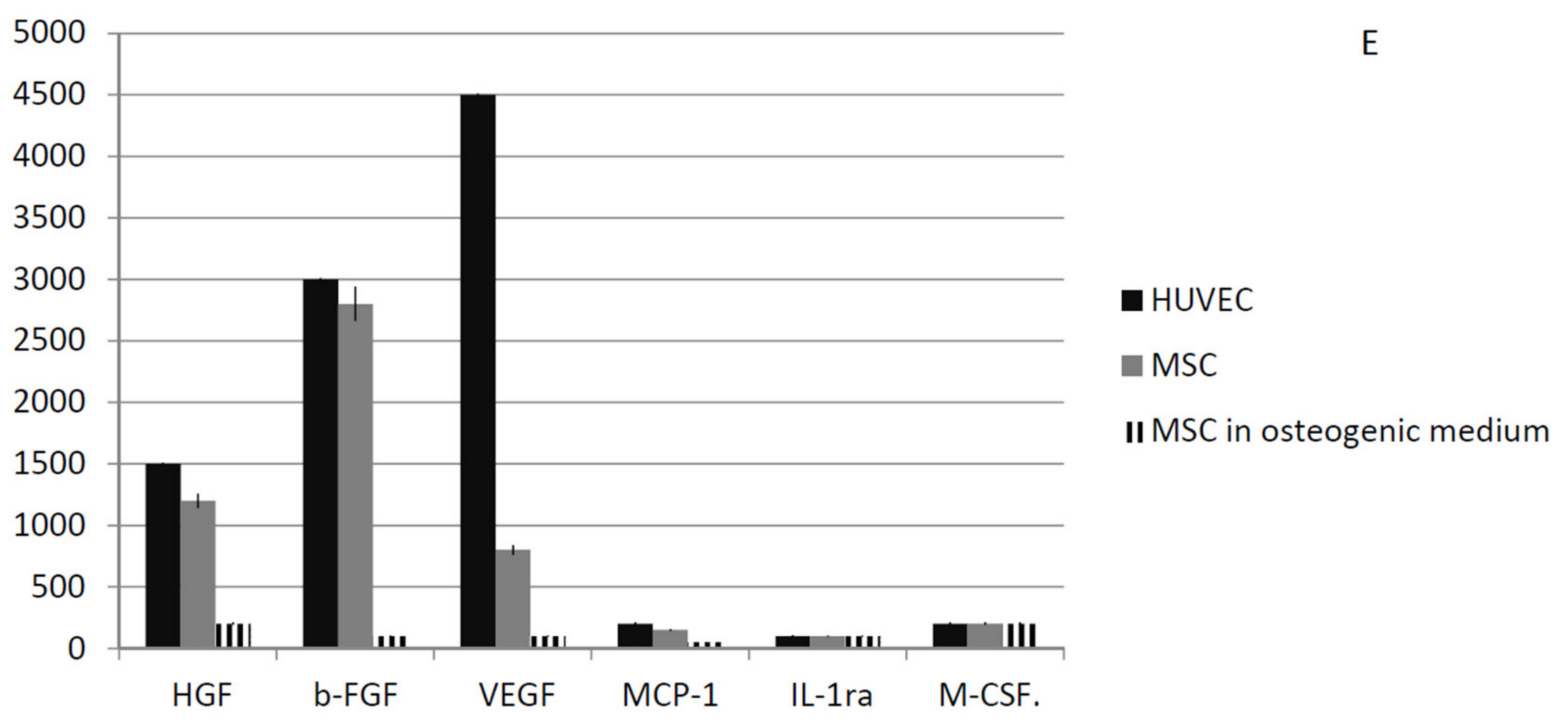
(**A**,**B**) SEM analyses of MSC in presence of no differentiative medium cultured onto OGI surfaces; (**C**) immunohistochemical test against phalloidine (red) (**D**) Quantification of growth factor release pg/mL. Statistically significant differences are indicated as ** *p* < 0.01; (**E**) Summarized graph about the quantification of growth factor release from the MSCs, osteoblastic like cells, and HUVEC cells.

**Figure 5:**
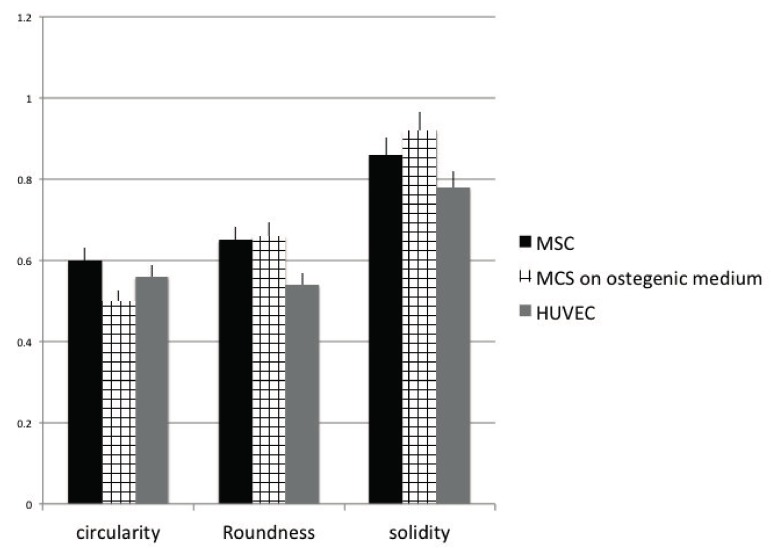
Analysis of the circularity, roundness, and solidity cell shape parameters after 14 days of culture onto OGI surfaces. MSC cultured in presence of nondifferentiative medium: (black bars), osteoinductive medium (squared bars); HUVEC (grey bars).

Mesenchymal stem cells cultured in a no differentiative medium Cells reached a continuous cell layer on day 14 (Figure 4A–C), with a confluent cell monolayer as SEM (Figure 5A,B) and positivity for phalloidine staining (red) (Figure 5C).

Several paracrine factors secreted by MSCs have been selected and detected by ELISA test are reported on Figure 5D where is evident that a good production of HGF, bFGF, and VEGF is present.

### 2.5. Cell Shape

In order to quantify the right phenotype of the cells we have performed an analyses of cells shape. We started from the assiom that endothelial cells have a small fusiform shape, osteoblastic cells a start-like phenotype, and MSC a fusiform shape. If cells maintain their right phenotype they will be able to exert their function on osteogenesis (osteoblastic and MSC activity) and angiogenesis (MSC and endothelial cell activity). Figure 5 is related to the analysis of cell shape. Focused has been related to three parameters: circularity, roundness, and solidity. The circularity and roundness calculated values confirm that when endothelial were seeded onto the OGI implants and cultured for 14 days, they assumed an elongated morphology. They also have the lesser solidity value. Osteoblastic cells acquire a less rounded morphology with the highest value on solidity. MSC has a more rounded aspect typical for a staminal phenotype.

### 2.6. ALP Activity

**Figure 6:**
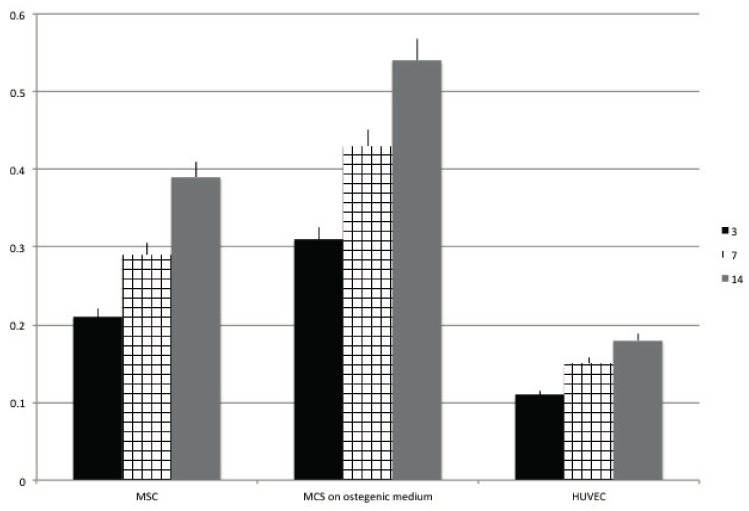
Analysis of ALP activity after 3 (black bars), 7 (squared bars), 14 (grey bars) days of culture onto OGI surfaces of MSC cultured in presence of nondifferentiative medium, osteoinductive medium; HUVEC

The alkaline phosphatase (ALP) activity of the cells seeded onto OGI surface was quantified and reported in Figure 6 in order confirm the possible early differentiation. Results confirm that ALP was higher when MSC where in pre osteoblastic like cell (0.25 U/mL) compare when cell are in a non-differentiative medium (0.24 U/mL) whether in HUVEC cells was slightly lower.

### 2.7. PCR

**Figure 7:**
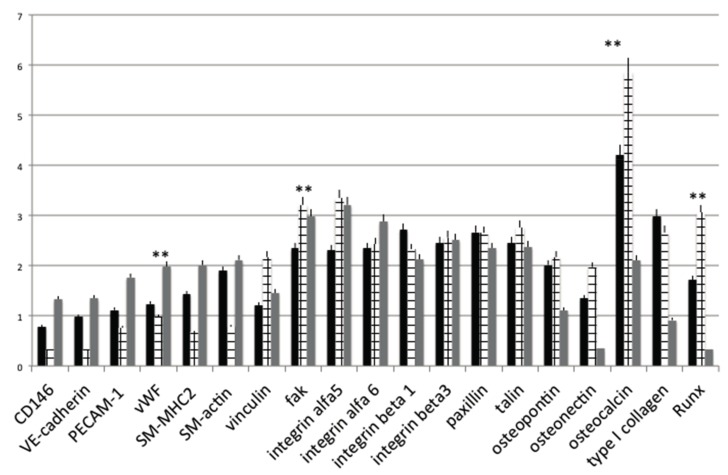
Gene expression profiles of osteogenic, cell adhesion molecules and endothelial differentiation markers in MSC cultured in presence of non differentiative medium: (black bars), osteoinductive medium (squared bars); HUVEC (grey bars.) seeded onto control or OGI surfaces. Values are expressed as 2^∆∆Ct^. Statistically significant differences are indicated as ** *p* < 0.01.

Moreover, the preservation or acquisition of endothelial cell phenotype on OGI surfaces was confirmed by the presence of angiogenic-osteogenic-cell adhesion endogenic cell-specific markers, at transcription levels using real-time RT-PCR (real time polymerase chain reaction), respectively, after cultured for 14 days on both control and OGI surfaces (Figure 7).

Our data show that the expression levels of angiogenic markers were higher on HUVEC compare in osteoblastic like cells. A well-defined expression is present on MSC cultures.

The osteoinductive properties of the OGI implants used in the present study were evaluated by means of the the gene expression level of osteoblast markers. The expression of selected genes (COL1A1, OCN, ON, OPN, RUNX) was evaluated about the expression of a reference gene (GAPDH) and compares the cultures on controls Titanium surfaces. As shown in Figure 7, the expression of some osteoblast markers in MSC in like osteoblastic trend is higher compared to the HUVEC and to a MSC control condition. In particular, high gene expression levels were observed for COL1A1, OPN, and RUNX.

The results related to the cell adhesion markers showed β1 and β3 integrin increase in osteoblastic differentiation markers on OGI surface. Moreover the same trend are present for integrin Alfa 5 and 6 that mediate adhesion of MSC cells to matrix proteins, and that are necessary for cell-matrix interactions in bone. Our results, confirm that in all cell types present in OGI surface gene expression of beta 1 and alpha 5 integrin are present. Osteoblasts uniformly expressed the alpha V.

## 3. Discussion

Cell-surface interactions could be considered as the primary factors of the long-term performance and bio functionality of dental devices since surfaces properties are able to promote osteoblast specific adhesion and enhance differentiation [2], increasing the exogenesis of mineralized matrix, bone tissue formation, and deposition. An adverse event such as fibrous encapsulation is further known to occur with both different surfaces [3]. This could reduce biocompatibility. However, OGI Titanium (Ti) surface microarchitecture has been shown to increase osteoblast differentiation [28,29,30,31,32] by our previous publication.

However, clinical success of implanted materials is related thanks to osteointegration and on neovascularization in the peri-implant bone. Here, we tested the hypothesis that OGI Ti surface is able to regulate up the expression and secretion of angiogenic growth factors on HUVEC cells, MSC in an undifferentiated state, and on a pre-osteoblastic feature have been seeded and analyzed on an OGI surface.

The values of secretion and expression ion of VEGF-A, and of FGF-2, confirmed the right endothelial activity of HEVEC when they are seeded onto an OGI surface. These factors were undetectable in osteoblastic-like cells cultures. These results show that the Ti surface modulates the secretion of angiogenic growth factors by osteoblasts. VEGF and b-FGF that are the most potent and widely investigated pro-angiogenic growth factors are produced by HUVECs when cultured on OGI surfaces in higher levels than those cells cultured on the control surface.

On this basis, we can conclude that OGI surfaces enhance the ability of HUVECs in producing angiogenic factors in vitro. This event has been moreover confirmed by the analyses of gene expression of other markers of the endogenic-associated genes, such as VWF, and PECAM-1. More over as revealed in Figure 7, position expression of VWF gene expression on the OGI surfaces suggest that HUVECs had some endothelial characteristics and functions after culturing on this surfaces. As evidenced from Figure 7, PECAM-1 expression on OGI surfaces was generally higher than that of the control surface, indicating increased endothelial cell proliferation and migration on the OGI surfaces, which is in good agreement with our cell proliferation results as shown in Figure 1. In order to evaluate the ability of the OGI surface to increase cells attachment of the “right cells” needed for osteointegration, an analyses of morphology and mediator of cell attachment was performed. The expressions of other markers related to cells attachment associated genes such as vincula, FAK, and integrin. The osteogenic influence of the surface has been in the end confirmed by the ability acquired by MSC to secreted typical osteoblastic factor such as HGF and MCSF by the production of ALP and by the gene expression related to osteogenic commitment such as osteopontin, osteonectin, and RUNKX. These data imply a better angiogenic and osteogenic potential of the as-grown OGI over the c control counterpart.

In a biomaterials setting, mesenchymal stem cells have been shown to undergo osteospecific differentiation and functional tissue formation when cultured on topographies that increase focal adhesion frequency and reinforcement. Our results confirm that on OGI surface MSC and osteoblastic like cells are able to increase focal adhesion and its related activation of RUNX pathway.

These results support the preclinical evidence of the good osteoproperties of OGI surface by Caroprese et al. [32] that analysed in the osseointegration of implants with OGI surface installed using a standard bed preparation in sites of different bone morphology. Results confirmed that the final insertion torque was 50–55 Ncm at the premolar and 30–35 Ncm at the molar sites. Mean osseointegration in percentage reached 61.5 ± 11.5% and 63.3 ± 10.1% at the premolar and molar sites, respectively. Mineralized bone density evaluated from the implant surface up to a distance of about 0.6 mm lateral to the implant surface was 63.0 ± 7.4% and 65.4 ± 17.7% at the premolar and molar sites, respectively. The authors conclude that similar implant bed preparations performed at premolar and molar sites with different bone morphology, yielding insertion torque values of about 30–35 and 50–55 Ncm, respectively, did not affect osseointegration after four months at non-submerged implants.

## 4. Materials and Methods

### 4.1. Dental Pulp Mesenchymal Stem Cells

Human dental pulps were extracted from healthy molar teeth, which had been extracted because of mucosal inflammation (impacted teeth with pericoronitis) or for orthodontic reasons from adult subjects aged 16 to 66. The pulps were classified into 6sixage groups (12 teeth per group). Each subject gave informed written consent for the use of his or her dental pulps. The Ethical Committee of Padua Hospital approved the research protocol. Before extraction, each subject was checked for systemic and oral infections or diseases. Only disease-free subjects were selected for pulp collection. Each subject was pretreated for one week with professional dental hygiene. Before extraction, the dental crown was covered with a 0.3% chlorexidine gel (Forhans, New York, NY, USA) for 2 min. After mechanical fracturing, dental pulp was obtained by means of a dentinal excavator or a Gracey curette. The pulp was gently removed and immersed for 1 h at 37 °C in a digestive solution: 100 U/mL penicillin, 100 mg/mL streptomycin, 0.6 mL of 500 mg/mL clarithromycin, 3 mg/mL type I collagenase, and 4 mg/mL dispase in 4 mL of 1 M PBS. Once digested, the solution was filtered through 70 mm Falcon strainers (Becton & Dickinson, Franklin Lakes, NJ, USA) [15].

### 4.2. Human Umbilical Vein Endothelial Cells (HUVEC)

HUVEC were prepared from Umbilical cords that were freshly supplied by the local Obstetric and Gynecology Division. Briefly, after perfusion of the vein with a metal gauge, the vein was rinsed twice with saline solution (PBS, phosphate buffered saline without Ca^2+^ and Mg^2+^). Endothelial cells were separated from the vein walls by collagenase type IA (Sigma, Kawasaki-shi, Japan) digestion (1 mg/mL; 300–400 U/mg) for 10 min at 371 °C in physiological solution. The reaction was terminated by adding complete Medium199 (Seromed, Istanbul, Turkey) (M199 plus 2 mm l-glutamine, 1/2100 U/mL; 1/2100 mg/mL penicillin/streptomycin, 2:5 mg/mL amphotericin B, i.e., cM199) supplemented with 20% FBS without growth factors. A maximum of three passages were allowed before seeding of endothelial cells into the OGI surfaces.

### 4.3. Cell Medium

Non-differentiative medium: Non-hematopoietic (NH) stem cell expansion medium (Miltenyi Biotec, Bergish Gladbach, Germany).

Osteogenic differentiation medium: NH OsteoDiff Medium (Miltenyi Biotec, Bergish Gladbach, Germany).

Endothelial differentiation medium: DMEM containing 10% FBS plus 0.1 ng/mL human recombinant ECGF, 10 mg/mL human bFGF (Calbiochem, San Diego, CA, USA) and 100 mg/mL porcine heparin (Seromed; Berlin, Germany).

### 4.4. MTT Test

Cell proliferation rates were determined by the MTT [3-(4,5-dimethylthiazol-2yl)-2,5-diphenyltetrazolium bromide)-based cytotoxicity test using the Denizot and Lang method with minor modifications [29].

### 4.5. DNA Content

DNA content was determined using a DNeasy kit (Qiagen, Hilden, Germany) to isolate total DNA from cell cultures following the manufacturer’s protocol for tissue isolation, using overnight incubation in proteinase K (Qiagen). The concentration of DNA was detected by measuring the absorbance at 260 nm in a spectrophotometer. Cell number was then determined from a standard curve (microgram DNA vs. cell number) generated by DNA extraction from counted cells. The standard curve was linear over the tested range of 5–80 µg DNA (r = 0.99) [30].

### 4.6. Growth Curve and Doubling Time

Cells were seeded into OGI dishes at an initial density of 5 × 104. When cells reached confluence, they were detached, counted and re-seeded at a density of 5 × 104. The PDT of the cells was calculated according to the formula:

PDT = (T − T_0_) log2/(logN_t_ − logN_0_) (1)

where PDT represents the cell doubling time, T represents the duration of cell culture, and N and N represent the cell number after seeding and the cell number after culturing for t hours, respectively [31].

### 4.7. Quantification of Secreted Factors

Secreted factors have been evaluated from culture supernatants. Cells (HUVEC, MSC; and osteoblast like cells) were seeded at a density of 50,000 cells/cm^2^ onto OGI surfaces. After 24 h, cells were washed twice with standard culture medium w/o FBS.

Hepatocyte growth factor (HGF), basic fibroblast growth factor (b-FGF), vascular endothelial growth factor (VEGF), monocyte chemotactic protein-1 (MCP-1), stromal cell-derived factor 1-alpha (SDF-1a), interleukin 1 receptor antagonist (IL-1ra), and macrophage colony-stimulating factor (M-CSF) were quantified using the respective Bio-Plex assay (Bio-Rad) according to manufacturer’s protocol.

### 4.8. Real-Time PCR

Human primers were selected for each target gene with Primer 3 software. Real-time PCRs were carried out using the designed primers at a concentration of 300 nM and FastStart SYBR Green Master (Roche) on a Rotor-Gene 3000 (Corbett Research, Sydney, Australia). Thermal cycling conditions were as follows: 15 min denaturation at 95 °C; followed by 40 cycles of 15 s denaturation at 95 °C; annealing for 30 s at 60 °C; and 20 s elongation at 72 °C. Differences in gene expression were evaluated by the 2^ΔΔCt^ method, using DPSCs cultured onto non treated titanium disks for 15 days as control. Values were normalized to the expression of the glyceraldehyde-3-phosphate dehydrogenase (GAPDH) internal reference, whose abundance did not change under our experimental conditions. Experiments were performed with three different cell preparations and repeated at least three times.

### 4.9. Scanning Electron Microscopy (SEM)

For SEM imaging, DPSCs grown on control and treated Ti surfaces for 15 and 25 days were fixed in 2.5% glutaraldehyde in 0.1 M cacodylate buffer for 1 h, then progressively dehydrated in ethanol. Control and treated Ti surfaces without cells were also examined. The SEM analysis was carried out at the Interdepartmental Service Center C.U.G.A.S. (University of Padova, Padova, Italy).

### 4.10. Statistical Analysis

One-way analysis of variance (ANOVA) was used for data analyses. The Leveneı’s test was used to demonstrate the equal variances of the variables. Repeated-measures ANOVA with a post-hoc analysis using Bonferroni’s multiple comparison was performed. *T*-tests were used to determine significant differences (*p*, 0.05). Repeatability was calculated as the standard deviation of the difference between measurements. All testing was performed in SPSS 16.0 software (SPSS Inc., Chicago, IL, USA) (license of the University of Padua, Padova, Italy).

### 4.11. ALP Activity Measurements

The alkaline phosphatase (ALP) activity was measured after 2 weeks of cell culture to evaluate the initial differentiation of MSC into preosteoblasts. Abcam’s Alkaline phosphates kit (colorimetric) has been used to detect the intracellular and extracellular ALP activity. The kit uses p-nitrophenyl phosphate (pNPP) as a phosphatase substrate which adsorbed at 405 nm when dephosphorylated by ALP. According to the manufacturer protocol, the culture medium from each sample group was collected and pooled together. At the same time, cells were washed with PBS and then homogenized with ALP Assay Buffer (300 μL in total for each group) and centrifuged at 13,000 rpm for 3 min to remove insoluble material. Different volumes of samples (medium and cells) were then added into 96-well plate, bringing the total volume in each well up to 80 μL with Assay Buffer. 80 μL of fresh medium was also utilized as sample background control. Thereafter, 50 μL of 5 mM pNPP solution was added to each well containing test samples and background control and incubated for 60 min at 25 °C, protecting the plate from the light. A standard curve of 0, 4, 6, 12, 16, and 20 nmol/well was generated from 1 mM pNPP standard solution bringing the final volume to 120 μL with Assay Buffer. All reactions were then stopped by adding 20 μL of Stop solution into each standard and sample reaction except the sample background control reaction. Optical density was read at 405 nm in a microplate reader (Victor). The results were normalized subtracting the value derived from the zero standards from all standards, samples and sample background control. The pNP standard curve was plotted to identify the pNP concentration in each sample. ALP activity of the test samples was calculated as follow:

ALP activity (U/mL) = A/V/T(2)

where:

A is the amount of pNP generated by samples (in μmol).
V is the amount of sample added in the assay well (in mL). T is the reaction times (in minutes).

### 4.12. Cell Shape Analyses

Cell area and different shape descriptors were calculated with ImageJ software (National Institutes of Health) (http://rsb.info.nih.gov/ij). In particular, the solidity (S), circularity (C), and roundness (R) of cells were investigated according to the following equations:

Roundness R = a/b(3)

where a and b are the width and length of the minimum bounding (the smallest rectangle enclosing the selection), respectively,

Circularity C = 4A/P2(4)

where P is the perimeter and A is the cell area, and

Solidity S = A/ConvexA(5)

where ConvexA is the area enclosed by the smallest shell that borders all the points of the cell.

### 4.13. Immunofluorescence

Cells were fixed in 4% paraformaldehyde in PBS for 10 min, then incubated in 2% bovine serum albumin (BSA, Sigma-Aldrich, Saint Louis, MO, USA) in PBS for 30 min at room temperature. Cells were then incubated with primary antibodies in 2% BSA solution in a humidified chamber for 12 h at 4 °C. The following primary antibodies were used: rabbit polyclonal antihuman phalloidine antibody (Millipore Corporation, Boston, MA, USA) and mouse polyclonal anti-human VEGF antibody (Sigma-Aldrich, Boston, MA, USA). Immunofluorescence staining was performed using the secondary antibodies DyLight 549-labeled anti-rabbit IgG (H + L) (KPL, Gaithersburg, MD, USA diluted 1/1000 in 2% BSA for 1 h at room temperature. Nuclear staining was performed with 2 μg/mL Hoechst H33342 (Sigma-Aldrich) solution for 2 min.
